# Characterization of a Neutral Sphingomyelinase Activity in Human Serum and Plasma

**DOI:** 10.3390/ijms24032467

**Published:** 2023-01-27

**Authors:** Christiane Mühle, Johannes Kornhuber

**Affiliations:** Department of Psychiatry and Psychotherapy, Universitätsklinikum Erlangen and Friedrich-Alexander-Universität Erlangen-Nürnberg (FAU), Schwabachanlage 6, D-91054 Erlangen, Germany

**Keywords:** ceramide, sphingomyelin, sphingolipid metabolism, enzyme inhibitors, blood biomarker, cobalt cofactor, NSM, EC 3.1.4.12, sphingomyelin phosphodiesterase, *SMPD3*

## Abstract

Alterations of sphingolipids and their metabolizing enzymes play a role in various diseases. However, peripheral biomarkers for such changes are limited. Particularly, in the increasingly reported involvement of neutral sphingomyelinase (NSM) with four described isoforms in tissues or cells, a peripheral marker is lacking. We here describe the detection of an NSM activity in human serum and plasma samples which hydrolyses fluorescently labeled sphingomyelin to ceramide in a time- and volume-dependent manner. Reaction rates were linear up to 10 days, and serum volumes above 2 vol-% were inhibitory. Biochemical properties were different from acid sphingomyelinase (ASM) with respect to detergent specificity (sodium deoxycholate), pH profile (pH 7–9), and cation dependence: Serum NSM activity was inhibited by EDTA ≥ 1 µM and restored in EDTA-anticoagulated plasma with the addition of ≥ 100 µM Co^2+^. It was independent of Mg^2+^, the typical cofactor of cellular NSM species, and even inhibited by [Mg^2+^] ≥ 20 mM. Serum NSM activity was not correlated with ASM activity and was independent of sex and age in 24 healthy adults. Since human peripheral NSM activity is very low and activities in rodents are even lower or undetectable, future research should aim to increase the reaction rate and determine the source of this enzymatic activity. The established activity could serve as a future biomarker or therapeutic target in diseases affected by sphingolipid derangements.

## 1. Introduction

Sphingomyelin is a major lipid in cellular and subcellular membranes of eukaryotic cells. Different sphingomyelinases (sphingomyelin phosphodiesterase, EC 3.1.4.12) catalyze the initial step in the catabolism of sphingomyelin, the hydrolysis of the phosphodiester bond of sphingomyelin to generate biologically active ceramide and phosphocholine. In contrast, sphingomyelin synthases catalyze the reverse reaction [1]. Ceramide can alter membrane fluidity or rigidity to mediate membrane dynamics and signaling molecules [2]. Thus, it is involved in intracellular signaling pathways such as cell proliferation, differentiation, and apoptosis and, as a lipophilic second messenger, plays a role in response to situations such as hypoxia, oxidative stress, and inflammation [3].

Sphingomyelinases differ in their tissue and subcellular distribution, regulation, and enzymatic properties, particularly their catalytic pH optimum: Lysosomal and secretory Zn^2+^-dependent acid sphingomyelinases (ASM) are encoded by the same gene, *SMPD1* [4]. A single alkaline sphingomyelinase (*ENPP7*) has been identified in the human intestinal tract and bile [5]. In contrast, there is a family of neutral sphingomyelinases (NSM). They can be classified into four types: NSM-1 (*SMPD2*), NSM-2 (*SMPD3*), NSM-3 (*SMPD4*), and a mitochondrial-associated NSM (MA-NSM, *SMPD5*) [6,7,8]. Although cloned first, overexpressed NSM-1 was found to lack an effect on sphingomyelin metabolism; rather, it efficiently hydrolyzed phosphatidylcholine and lyso-platelet-activating factor. NSM with a hydrophobic C-terminus resides in the nucleus and endoplasmic reticulum and is Mg^2+^-dependent [9]. An NSM 1-deficient mouse model lacked any obvious phenotype or lipid storage abnormalities but also showed unperturbed lyso-platelet-activating factor levels [10]. The second cloned enzyme, NSM-2, possessed a Mg^2+^-dependent activity affecting sphingomyelin and ceramide levels in vitro and in vivo [11]. NSM-2 is mainly localized to the Golgi apparatus and plasma membrane. NSM-2 knockout mice do not have a deficiency in lipid storage but unexpectedly develop growth retardation as embryos [12]. NSM-2 is currently the most studied isoform with established roles in bone mineralization, cell growth arrest, exosome formation, and the inflammatory response. The ubiquitously expressed NSM-3 shares negligible homology with other NSMs but is also Mg^2+^-dependent and localizes to the Golgi apparatus and endoplasmic reticulum [13]. Finally, MA-NSM was only cloned in 2010 based on its significant homology to NSM-2 [7]. The functional enzyme MA-NSM localized predominantly in mitochondria and shows an absolute requirement of cations such as Mg^2+^ and Mn^2+^ and activation by anionic phospholipids.

The sphingomyelinase most active at an acidic pH, ASM is ubiquitously expressed and has been well characterized biochemically and in a wide range of diseases [14,15,16]. Lysosomal ASM cannot only be detected in tissue and cell lysates but also in peripheral blood mononuclear cells. Moreover, secretory ASM expressed constitutively from the same gene can be measured in human serum and plasma as well as cerebrospinal fluid [17]. However, comparable knowledge for NSM is lacking. Due to its up to 20-fold higher expression in the brain compared to peripheral tissues [18], this enzyme could be of increased interest for neuroscience. Indeed, NSM-2 has been involved in the biogenesis of exosomes and the release of extracellular vesicles with relevance to many conditions, from cardiovascular [19] and cardiometabolic [20] diseases [19] to anxiety [21], major depressive disorder [22], and alcohol addiction [23,24]. While different tissue types are easily available from animal models, including brain regions, biological material from patients is mostly limited to peripheral liquid biopsies such as easily available blood, saliva, or urine samples, whereas tissue biopsies require more invasive procedures and may pose ethical problems. With the advancement of the use of circulating ceramides as biomarkers and potential metabolic and other disease treatments [25], specific enzymatic activities would complement the approach of personalized medicine and more clearly indicate the source of alterations or potential druggable targets.

With this increased interest in NSM but limited availability of samples from patients, we aimed to determine whether NSM would be detectable in human blood, to optimize a quantitative assay, and to characterize the biochemical properties with the prospective of an application as a biomarker.

## 2. Results

### 2.1. Presence of NSM Activity in Human Serum: Volume and Time Dependence

**Figure 1:**
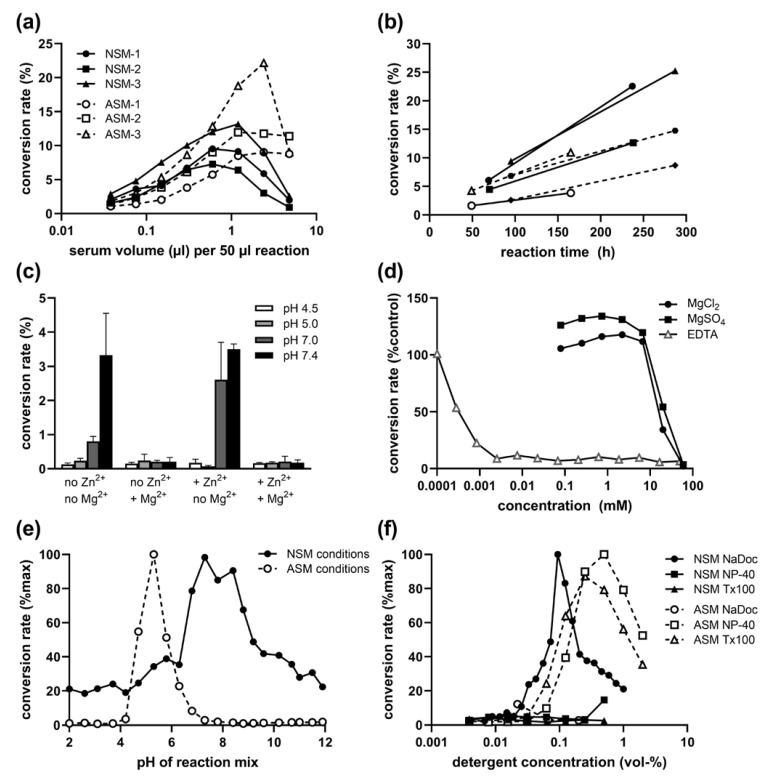
Optimization of reaction conditions for NSM in human serum samples. (**a**) Sample volume dependence for three representative samples (1–3) for ASM (16 h reaction time) and NSM (100 h). (**b**) Time dependence of serum NSM in different samples. (**c**) pH-dependent influence of 500 µM Zn^2+^ and 200 mM Mg^2+^ addition on ASM and NSM activity (reaction mix with 0.1% NaDoc). (**d**) Inhibition of serum NSM activity by Mg^2+^ and EDTA. (**e**) pH profile for ASM (NP-40, Zn^2+^) and NSM (NaDoc) conditions. (**f**) Influence of detergents for ASM and NSM conditions. NaDoc sodium deoxycholate, NP-40 Nonidet P-40, and Tx100 Triton X-100 detergents.

In a reaction mix containing sodium deoxycholate (NaDoc) as a detergent, we were able to detect low NSM activity in human serum samples, which was not measurable before using our standard NSM assay for tissue or cell lysates containing Nonidet P-40 (NP-40). The hydrolysis of fluorescently labelled sphingomyelin to ceramide at a neutral pH was volume-dependent (Figure 1a). It increased nearly linearly and stably over time (Figure 1b) with good reproducibility despite the very low activity, which requires incubation over several days compared to hours for ASM in serum, or even shorter times for NSM in rodent brain lysates.

Serum and plasma show an inhibitory effect on the ASM reaction at overly high volumes (Figure 1a), limiting the utilized sample volume to about 1 µL per 50 µL reaction. We therefore tested the influence of the sample volume on NSM and found a slightly stronger inhibitory effect (Figure 1a). Thus, 0.6 µL per 50 µL reaction were used for further assays. This difference is also an indication that the detected NSM activity is not a residual ASM activity at a neutral pH.

### 2.2. Inhibitory Effect of Mg^2+^ on Serum NSM Activity

To further exclude the possibility that we are detecting residual secretory ASM activity under neutral pH conditions, we tested the effect of Zn^2+^, ions which are required for serum ASM activity at acidic and neutral pH (under NSM conditions with NaDoc detergent) and did not detect any increase in activity (Figure 1c). Interestingly, the activity was also not increased by the addition of 200 mM MgCl_2_, the optimal concentration for NSM activity assays in tissue lysates. In contrast, NSM activity was only detectable in absence of MgCl_2_ (Figure 1c). Therefore, the detected enzymatic activity hydrolyzing sphingomyelin to ceramide at neutral pH could not be the most commonly analyzed NSM-2 (gene *SMPD3*) Mg^2+^-dependent species or other known NSM family members described to be Mg^2+^ dependent [26].

Indeed, concentrations of both MgCl_2_ and MgSO_4_ above 7 mM in the reaction mix strongly inhibited the NSM activity from serum samples, while there was no clear beneficial effect at lower concentrations. However, similar to tissue NSM activity, serum NSM activity was also strongly reduced by the presence of ethylenediaminetetraacetic acid (EDTA, at concentrations above 1 µM, Figure 1d), a reagent chelating divalent cations such as Mg^2+^. Thus, the serum NSM enzymatic activity appears to depend on a different divalent cation.

### 2.3. pH and Detergent Profile of Serum NSM Activity

Further verification of a serum NSM activity independent of any residual serum ASM activity was provided by the clearly different pH profile when using enzyme-specific detergents and Zn^2+^ for ASM (Figure 1e). The highest ratio of NSM/ASM activity was found in pH 8.4 and 8.8, representing specifically NSM activity, and lowest ratio in pH 4.7–5.8, representing most specifically the ASM peak of activity. This pH profile for NSM with a broad optimum of pH 7–9 was also replicated with non-phosphate buffers (sodium acetate, HEPES, and Tris/HCl).

Additional evidence for different enzymes stems from the contrary preference for detergents: While ASM is active with both NP-40 and Triton X-100 (Tx-100) with optimal concentrations around 0.25 to 1%, and shows no activity with NaDoc, the opposite is true for NSM: it is active only with NaDoc (0.03 to 0.3%) but not with NP-40 or Tx-100 (Figure 1f).

### 2.4. Kinetics of Serum NSM Activity

**Figure 2:**
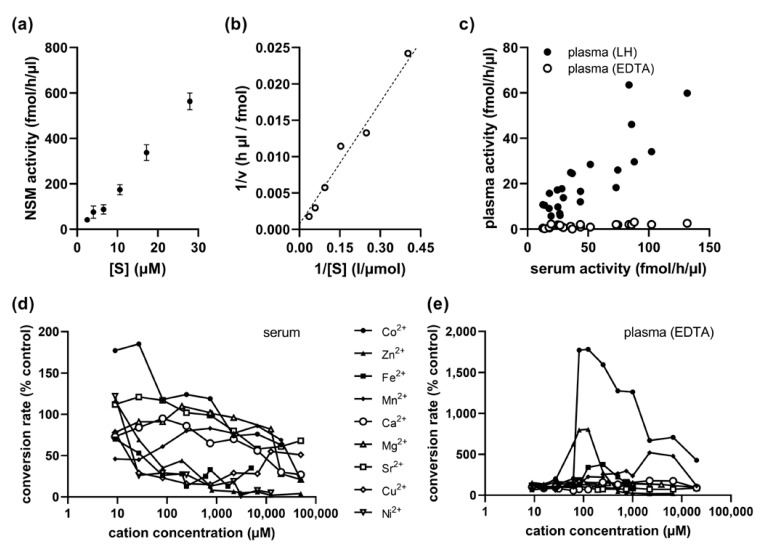
Kinetics and optimization of reaction conditions for EDTA-anticoagulated plasma samples. (**a**) Substrate–velocity graph for serum NSM activity. (**b**) Lineweaver–Burk plot with nonlinear regression line. (**c**) Correlation between NSM activities in serum and lithium heparin (LH) anticoagulated plasma versus nearly undetectable NSM activities in EDTA-anticoagulated plasma. (**d**) Mostly inhibitory influence of different divalent cations on NSM activity in serum samples. (**e**) Influence of cations on NSM activity in EDTA-anticoagulated plasma samples with the highest benefit from addition of cobalt ions.

A nearly constant percentage rate of conversion from sphingomyelin to ceramide for substrate concentrations up to 28 µM indicated that the standard assay concentration of 0.6 µM labeled sphingomyelin is far below the saturation limit (Figure 2a). Non-linear regression analysis using the classical Michaelis–Menten model yielded an apparent K_m_ value of 60 µM. Therefore, a substrate concentration of ideally over 10 times the K_m_ value for a saturated assay is not realistic for routine analysis due to economical and practical reasons. There was no evidence of cooperativity based on the non-sigmoidal shape of the substrate–velocity graph. The reaction followed Michaelis–Menten kinetics with a maximum activity (V_max_) of 1500 fmol/h/µL (Figure 2b).

### 2.5. Cobalt Cation Dependence of NSM in EDTA-Anticoagulated Plasma

While NSM was detectable in serum and also in plasma samples anticoagulated with lithium heparin and showed a very high correlation (r = 0.850, *p* < 0.001, *n* = 24), there was nearly no activity in plasma samples anticoagulated with EDTA (Figure 2c). This was well in agreement with the inhibitory effect of added EDTA observed in serum samples (Figure 1d), since EDTA vials contain approximately 1.5–2 mg EDTA/mL of blood, resulting in a final concentration of around 50 µM EDTA in the reaction. We therefore tested the addition of various divalent cations to the reaction of serum NSM to identify a candidate with a positive effect, but found mostly inhibitory activities, except for low concentrations of cobalt (Figure 2d). Indeed, the addition of Co^2+^ cations at concentrations above 100 µM resulted in an increase in NSM activity from EDTA-anticoagulated plasma by more than 10-fold, while most other cations showed no effect, except for Zn^2+^, which could possibly result from some low residual ASM activity during long incubation times, and Mn^2+^ (Figure 2e).

### 2.6. No Association of Serum NSM with Sex, Age, or ASM Activity

Comparing serum NSM activities between female and male healthy controls (*n* = 12 each) revealed no significant sex difference (*p* = 0.073). Moreover, no correlation of serum NSM activity with age was observed (*p* = 0.692, *n* = 24, range 32–67 years).

Furthermore, the independence of ASM and NSM activities in serum was supported by the lack of correlation of both activities in healthy adults (*p* = 0.955, *n* = 24). In these individuals, the absolute activity (fmol/h/µL serum) for ASM was 4- to 20-fold (mean 9-fold) higher than for NSM.

### 2.7. Very Low to Undetectable Serum NSM Activities in Rodents

To apply the established assay for animal models as well, we tested mouse and rat serum samples for NSM activity. Surprisingly, serum NSM activity was extremely low, almost undetectable, in wild-type mice, with a 10-fold lower level compared to human serum. In rat samples, we were not able to detect any ceramide product even after prolonged incubation. In contrast, secretory ASM activities were easily measurable in mouse serum, with activities comparable to humans, and in rat serum, with about 6-fold higher ASM activities.

## 3. Discussion

Particularly, NSM has gained much attention as its role in sphingolipid alterations in a range of diseases is discovered, yet a peripheral activity in easily available human blood samples has not been reported so far, to our knowledge. We here describe for the first time the presence of NSM activity in human serum and plasma samples with distinct biochemical properties and its potential applications as a biomarker or therapeutic target. The optimized assay resulted in a reliable volume- and time-dependent hydrolysis rate of sphingomyelin to ceramide at neutral pH, but activities were very low and required long incubation times of several days. It would, thus, be desirable to further optimize the assay by testing different detergent types, other substrates such as NBD-labeled or radioactively labeled sphingomyelin, or the addition of sphingolipids, unsaturated fatty acids (known to activate NSM2, [27]) or taurocholate (known to activate alkaline sphingomyelinase [28]), or the generation of micelles to enhance the reaction rate. Moreover, the utilized labeled C12-sphingomyelin might not present the optimal substrate, and other fatty acid chain length species should be tested either as labeled sphingomyelins or in a competitive assay with an excess of unlabeled sphingomyelin species of different fatty acid chain lengths.

Based on the literature and our own experimental data presented here, we can exclude the possibility that the detected serum NSM activity originates from residual ASM activity at neutral pH for a number of reasons: the distinct pH peak, the opposite detergent preference, the different portion of serum inhibiting the reaction, and the lack of a correlation of serum ASM and NSM activities.

We studied the available literature to argue whether the observed serum NSM activity could be attributed to alkaline sphingomyelinase with an optimal pH of 8.5–9.0 and a range of activity between pH 7 and at least 10 for the purified [29] and recombinant [30] enzymes largely overlapping the broad pH optimum 7–9 found for serum NSM. Alkaline sphingomyelinase is reported to be present only in the intestinal tract and human bile and is responsible for the digestion of dietary sphingomyelin [31]. In contrast to the rapid inactivation of ASM and NSM by pancreatic trypsin [5,32], the secreted C-terminally truncated form of alkaline sphingomyelinase is released by pancreatic trypsin and is more active than the membrane-bound enzyme at the surface of microvilli [33]—thus, there is a soluble active isoform. Although its main substrate is sphingomyelin, it is also able to cleave lysophosphatidylcholine and inactivate the signaling lipid platelet-activating factor [30]. The activity of alkaline sphingomyelinase is dependent on bile salts (a class of physiological anionic detergents): it is very low in their absence and reaches a maximum at the critical micelle concentration of each bile salt [5]. This resembles the strong biphasic dependence of serum NSM on NaDoc (sodium deoxycholate, i.e., a bile salt used as ionic detergent) and no effect of other detergents such as NP-40 or Triton X-100. Similarly, Triton X-100 did not activate alkaline sphingomyelinase [32]. Alkaline sphingomyelinase shares no structural similarity with other sphingomyelinases and belongs to the nucleotide pyrophosphatase/phosphodiesterase (NPP) family, and is therefore also called NPP7. While retaining conserved amino acid residues forming the two metal-binding sites in NPP, its sphingomyelin hydrolyzing activity is not stimulated by Ca^2+^ or Mg^2+^ but inhibited by Zn^2+^, in contrast with most other NPP family members [5]. Serum NSM was also insensitive to low concentrations of Ca^2+^ and Mg^2+^ but not inhibited by Zn^2+^. Moreover, Cu^2+^ had no effect on intestinal alkaline sphingomyelinase up to 50 µM [32], but markedly reduced serum NSM activity. Data on Co^2+^ are lacking. However, alkaline sphingomyelinase is not inhibited by EDTA [28], and routine assays even contain 2 mM EDTA [34,35], whereas serum NSM is strongly inhibited by EDTA concentrations above 1 µM—the most striking disparity. Taken together, despite many shared characteristics of the soluble alkaline sphingomyelinase, there are also clear differences to the detected NSM in serum, most prominently the sensitivity to EDTA, pointing towards a distinct enzymatic activity.

The only report found on NSM activity in human liquid biopsies versus cell/tissue lysates is the detection and purification of NSM from urine in 1989 [36]. However, the authors already speculate about the origin of this magnesium-dependent NSM from kidney proximal tubular cells. The shedding of these cells in human urine has been demonstrated and a monospecific polyclonal antibody immunoprecipitated both the purified urinary and the membrane-bound NSM from proximal tubular cells. Another peripheral membrane-bound NSM activity activatable by Mg^2+^ was found in chicken erythrocytes but not in human erythrocytes [37]. Therefore, an important question remains in determining the source of the NSM activity and the identity of the enzyme(s) with this activity in blood. Although judged unlikely, we cannot exclude the possibility that the measured sphingomyelinase activity at neutral pH stems from several new enzyme species or contains major contributions from the known NSM family members. In addition, the portions of activity of different NSM species might vary between samples from healthy controls and patients, such as alcohol-dependent individuals used in this investigation. However, we observed the same biochemical characteristics, including similar K_m_ and V_max_ values for control and patients’ samples, in all experiments. Four isoforms, NSM-1, 2, and 3, and MA-NSM have been described so far. Such functional multiplicities for enzymes seem reasonable from a cellular perspective as they allow for different localizations to specific compartments and separate regulatory mechanisms, as well individual substrate specificities. Since the NSM activity described here is Mg^2+^ independent, whereas the four known species seem to require Mg^2+^, the origin of serum NSM activity appears elusive. Furthermore, all mammalian NSM species contain an extra hydrophobic domain that tethers the catalytic domain to the membrane [26]. Thus, they are not assumed to be present in a secreted form in the blood unless some cleavage gives rise to a soluble but still active residual protein.

The dependence on Co^2+^ instead of Mg^2+^ as the typical cation cofactor in mammalian NSM family members was very surprising. Additionally, the yeast homolog of NSM was found to depend on the presence of Mg^2+^ [38]. In contrast, the presence of the usual Mg^2+^ concentration applied for tissue lysate assays was even inhibitory for serum NSM. Most other cations seemed to exert an inhibitory effect. However, there was a limitation to applying higher concentrations of some cations due to an interference with the BODIPY fluorescence on the chromatography plates. There are two old reports on Mg^2+^-independent NSM isoforms: An NSM of low activity and poor stability with 45 and 95 kDa peaks was identified in the cytosol of the human leukemia cell line HL-60 [39]. It was not activated by Mg^2+^ and not inhibited significantly by 0.5 M EDTA. Another Mg^2+^ (5 µM) insensitive 53 kDa NSM was found in rabbit skeletal muscle fractions together with a similarly active Mg^2+^ sensitive (activity increase by 43%) 92 kDa isoform. Due to affinity to the same antibody and a homologous peptide pattern after partial proteolytic digestion, the authors assumed that the 53 kDa protein is a proteolytic product or splice variant derived from the 92 kDa NSM [40]. Follow-up data including the effect of Co^2+^ are lacking. It thus remains to be verified whether the detected serum NSM activity could originate from this enzyme released into the blood. A comparable metal ion activation of sphingomyelinase activity to that observed in serum NSM has been reported for sphingomyelinase from *Bacillus cereus*, a homologue of mammalian NSM, in the order Co^2+^ ≥ Mn^2+^ ≥ Mg^2+^ » Ca^2+^ ≥ Sr^2+^ [41], which to some degree resembles the observations for serum and plasma NSM here, with respect to the applied concentrations of 1 to 5 mM. The discussed crystal structure of *B. cereus* sphingomyelinase with bound Mg^2+^ or Co^2+^ could provide some framework applicable to phosphohydrolases belonging to the DNase I-like folding superfamily, such as NSM. Moreover, the activation of *B. cereus* NSM by low Zn^2+^ concentrations (µM) and its inhibition by higher Zn^2+^ concentrations (mM), attributed to the binding of the cation to the high vs. low affinity binding sites of the enzyme [42], was also shared by the serum NSM activity. The apparent K_m_ value of 60 µM found for serum NSM activity was similar to the K_m_ values reported for sphingomyelinase from *B. cereus*, which were determined, using [*N*-methyl-^14^C]sphingomyelin, to be in a range of 14–19 µM, depending on the bound divalent metal ion [41]. It was also comparable to the apparent K_m_ of 83 µM for Mg^2+^-dependent neutral sphingomyelinase from rat brain microvessels and 61 µM from rat forebrain homogenate [43]. Interestingly, the K_m_ was also close to the range of 20–77 µM reported for ASM from different sources [17].

Due to the observed extremely low activity in mouse serum, checking knockout or transgenic animals for NSM to verify the assay in the case of the corresponding gene will most likely not be helpful. However, beyond the scope of this project, it would be interesting to compare serum NSM activities in different species other than rodents reported here.

Determining a profile of inhibition for serum NSM by a number of pharmacological inhibitors in comparison to ideally cloned isoforms of NSM could aid in the elucidation of the identity of serum NSM. For example, glutathione (GSH), an endogenous inhibitor of NSM-2 [44], GW4869 as a reportedly specific non-competitive inhibitor of NSM [45], DPTIP as a potent non-competitive NSM-2 inhibitor [46], or phytosphingosins as apparent competitive inhibitors of NSM [47] could be applied in serial dilutions to the enzymatic assays to determine half maximal inhibitory concentrations. Further drugs such as scyphostatin, C11AG, and manumycin A have been reported to inhibit NSM activity and could be applied [48]. Since most of these substances have not been extensively characterized for recombinant NSM isoforms, utilizing siRNAs specific to these NSM species could be of help in a subtractive approach. Moreover, determining serum NSM activity after inactivation or immunoprecipitation of different NSM species via specific antibodies could aid in the identification process. Additionally, comparing profiles of substrate specificity and activation factors of NSM species would provide a further approach.

Altered sphingolipid metabolism has been found in a wide range of diseases, including depression and alcohol dependence [18]. In these cases, we can also expect to observe adaptations of further enzymes such as NSM to counteract increased ceramide levels. It would, thus, be beneficial to be able to assess peripheral markers, such as the serum NSM activity established in this project. Further work is needed to increase the reaction yield of the assay. For routine application, additional properties of this enzymatic activity should be determined, including stability, which influence pre-analytical processes such as blood drawing (type of vial, requirement of protease inhibitors, and rapid cooling), transport, and storage conditions. Once the source of the enzymatic activity, i.e., the corresponding gene(s), has been determined, analysis of the promoter(s), splice variants, and regulation could shed further light onto the complex sphingolipid pathway processed similarly as observed for the splice variants of the ASM gene *SMPD1* [49], and their alteration in patients with major depressive disorder [50,51]. A better understanding underlying NSM and ceramide regulation is a prerequisite for future applications in a preventive or therapeutic setting [8].

## 4. Materials and Methods

### 4.1. Blood Samples

We used dispensable blood samples from the Neurobiology of Alcoholism (NOAH) study [52] from individuals with larger sample volumes. The NOAH study was conducted in accordance with the Declaration of Helsinki and approved by the Ethics Committee of the Friedrich–Alexander University Erlangen–Nürnberg (ID 81_12 B). Blood was collected from healthy controls and alcohol-dependent patients in the morning. Serum and plasma (with lithium heparin or EDTA anticoagulant) were centrifuged for 10 min and 2000× *g* at room temperature. Serum and plasma were then aliquoted and placed into storage at −80 °C for later analysis.

### 4.2. ASM and NSM Activity Assay

The activity of ASM and NSM was determined using the fluorescent substrate BODIPY-FL-C12-sphingomyelin (D-7711, Thermo Fisher Scientific, Schwerte, Germany) with four replicates each as described previously [53], except for the modification of the reaction mix. A standard reaction contained 29 pmol substrate in a total volume of 50 µL and was initiated by the addition of 6 µL of serum or plasma sample prediluted to 1:10 in 154 mM physiological NaCl solution. Optimal conditions for the reaction mix were 200 mM sodium acetate buffer (pH 5.0) with 500 mM NaCl, 0.02% Nonidet P-40 detergent, and 500 µM ZnCl_2_ for serum ASM and 200 mM HEPES (pH 7.35), 500 mM NaCl, and 0.1% NaDoc detergent for serum NSM, respectively.

For optimization of reaction conditions, a series of Carmody buffers (mixtures of 0.2 M boric acid, 0.05 M citric acid, and 0.1 M trisodium phosphate) adjusted to pH 2–12 [54] or Tris/HCl buffer, the variation of detergents (including Triton X-100), or supplementation with various chloride salts of divalent cations were tested. Reactions for kinetic analysis were performed with 0.2 µL serum in 20 µL with varying substrate concentrations and a constant amount of the substrate solvent dimethyl sulfoxide. After incubation for different times at 37 °C, reactions were analyzed directly or stopped by freezing at −20 °C and stored until further processing.

For analysis, 1.5 µL reaction mix was spotted on silica gel 60 thin layer chromatography plates (818232, Macherey-Nagel, Düren, Germany). Ceramide and uncleaved sphingomyelin were separated over a distance of 2.5 cm using a mixture of ethyl acetate with 1% (*v*/*v*) acetic acid as a solvent and were quantified on a Typhoon Trio scanner (GE Healthcare; 488 nm excitation, 520 nm emission, 325 V, 200 µm resolution) with the QuantityOne software (Bio-Rad Laboratories, Feldkirchen, Germany). Enzymatic activity is presented as the hydrolysis rate of sphingomyelin (fmol) per time (h) and per sample volume (µL) for individual samples. For optimization and characterization assays, the absolute conversion rate (percentage of sphingomyelin substrate converted to ceramide product) is given or this rate is normalized to that of an untreated control or to maximum levels.

### 4.3. Statistics

We used IBM SPSS for Windows 28.0 (SPSS Inc., Chicago, IL, USA) and Graph Pad Prism 9 (Graph Pad Software Inc., San Diego, CA, USA) for statistics and visualization and report mean values of quadruplicates for pooled samples (optimization/characterization) or for individual samples. Kinetic parameters V_max_ and K_m_ were calculated by nonlinear regression using the Michaelis–Menten model in Graph Pad Prism 9. Because the enzyme activities deviated significantly from normal distribution according to the Kolmogorov–Smirnov test, non-parametric methods were employed, i.e., the Mann– Whitney U test for group differences and Spearman correlations, respectively. *p* < 0.05 for two-tailed tests was considered significant.

## 5. Conclusions

In summary, we here describe—to our knowledge—for the first time an NSM activity present in human serum and plasma with an optimized assay and characterization of biochemical properties different from ASM, including a dependence on the divalent cobalt cation in contrast to the NSM-typical dependence on Mg^2+^. These data provide a new sphingolipid enzymatic activity and an assay for its routine assessment and evaluation as a potential biomarker and therapeutic target in diseases with aberrant sphingolipid metabolism. We also point towards further work required mainly to determine the source and genetic origin of this enzymatic activity and to estimate its clinical significance.

## Data Availability

Data are available upon request.
